# Feasibility of Fetal Portal Venous System Ultrasound Assessment at the FT Anomaly Scan

**DOI:** 10.3390/diagnostics12020361

**Published:** 2022-01-31

**Authors:** Rodica Daniela Nagy, Dan Ruican, George-Lucian Zorilă, Anca-Maria Istrate-Ofiţeru, Anne Marie Badiu, Dominic Gabriel Iliescu

**Affiliations:** 1 Doctoral School, University of Medicine and Pharmacy of Craiova, 200349 Craiova, Romania; rodica.nagy25@gmail.com (R.D.N.); ruican.dan@hotmail.com (D.R.); 2 Department of Obstetrics and Gynecology, University Emergency County Hospital, 200642 Craiova, Romania; ancaofiteru92@yahoo.com (A.-M.I.-O.); dominic.iliescu@yahoo.com (D.G.I.); 3 Department of Obstetrics and Gynecology, University of Medicine and Pharmacy of Craiova, 200349 Craiova, Romania; 4 Department of Histology, University of Medicine and Pharmacy of Craiova, 200349 Craiova, Romania; 5 Research Centre for Microscopic Morphology and Immunology, University of Medicine and Pharmacy of Craiova, 200349 Craiova, Romania; 6 Department of Pathology, University Emergency County Hospital, 200642 Craiova, Romania; anne.marie.khatib@gmail.com; 7 Department of Pathology, University of Medicine and Pharmacy of Craiova, 200349 Craiova, Romania; 8 Ginecho Clinic, Medgin SRL, 200333 Craiova, Romania

**Keywords:** portal venous system, fetal abnormalities, FT ultrasound, ultrasound, autopsy, immunohistochemical

## Abstract

Objective: To investigate the feasibility of the first trimester (FT) ultrasound scan (US) for the evaluation of the fetal portal venous system (PVS) anatomy, and to evaluate the potential of microcopy for a proper pathology evaluation for the PVS in the FT. Methods: We evaluated the PVS in 200 scan examinations performed in FT pregnancy. Half of the cases were scanned by two operators with extensive experience in obstetric ultrasound—Group I, and the other half was evaluated by two sonographers with less experience—Group II. Second-trimester US and autopsy in terminated pregnancies were used as follow-up. The pathologic evaluation was supported by microscopy. Results: all PVS features were successfully assessed by transabdominal ultrasound (TAUS) in 27% of the Group I cases and 14% in Group II. These rates increased to 88% in Group I and in 72% in Group II, after rescanning and using transvaginal ultrasound (TVUS). The conditions that led to rescanning and TVUS were: BMI greater than 24 in 26% cases, unfavorable fetal position (12.32%), retroverted uterus (12.32%), abdominal scar (10.96%), fibroids (4.11%), and combinations of the above (34.23%). The L-shaped UV confluence was identified transabdominally in 91% in Group I and in 79% in Group II and increased to 98% and 95%, respectively, following reevaluations. Microscopy represented a useful audit in all FT investigated cases. Conclusions: At the end of the FT, the visualization of a normal L-shaped UV confluence, that excludes major PVS abnormalities, is achievable in approx. 80%, indifferently the examiners experience. The sonographers experience, pregnant women BMI, and uterine anomalies as fibroids or retroversion significantly affect the rate of visualization, and necessitates vaginal approach and reexamination. The FT pathology, the audit of the ultrasound findings can only be performed microscopically, with relatively little resources involved and good results.

## 1. Introduction

**Figure 1:**
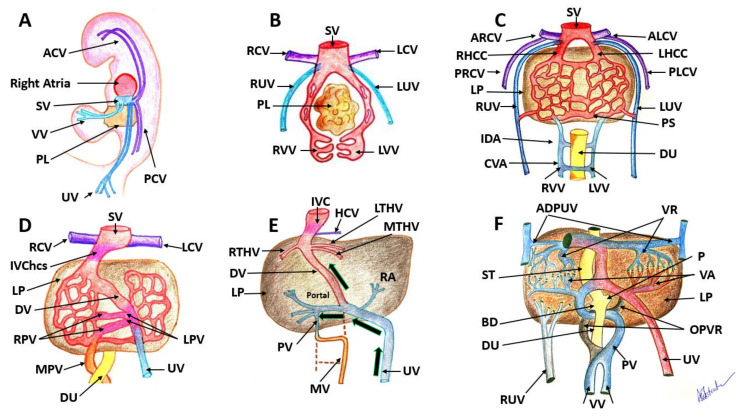
Embryological development of the human venous system. (**A**): The embryo demonstrates the development of paired sets of ‘vitelline’ and ‘umbilical’ veins in its fifth week, which initially drain the yolk sac and allantois. (**B**): At 4 weeks there are three symmetric paired veins: the umbilical veins, vitelline veins and cardinal veins. All three systems converge into the sinus venosus. (**C**): Liver cords develop into the septum transversum and interrupt the cranial portion of the umbilical and vitelline veins. (**D**): this is an asymmetric stage with the intrahepatic anastomosis between umbilico–portal and –ductus venosus systems. (**E**): Changes in the VVs and changes in the UVs continue. Only the most caudal and the most cranial segments of this portion of the VVs will persist. (**F**): Small venous branches, named venæ advehentes, convey the blood from the subhepatic anastomosis to the sinusoidal plexus; small venous vessels named venæ revehentes, drain the blood of the sinusoidal plexus into the subdiaphragmatic anastomosis. SV: sinus venosus, VV: vitelline veins, PL: primordial liver, UV: umbilical veins, PCV: posterior cardinal veins, ACV: anterior cardinal veins, RUV: right umbilical vein, LUV: left umbilical vein, RVV: right vitelline vein, LVV: left vitelline vein, ARCV: anterior right cardinal vein, ALCV: anterior left cardinal vein, DU: duodenum, PS: portal sinus, IDA: intermediate dorsal anastomosis, CVA: caudal ventral anastomosis, PRCV: posterior right cardinal vein; PLCV: posterior left cardinal vein; RHCC: right hepatic common cardinal vein; IVChcs: inferior vena cava hepatocardiac segment; RPV: right portal vein; LPV: right portal vein; UV: umbilical vein; MPV: main portal vein; LP: liver parenchyma; IVC: inferior vena cava; PV: portal vein; MV: mesenteric vein; RA: ramus angularis; RTHV: right terminal hepatic vein; MTHV: median terminal hepatic vein; LTHV: median terminal hepatic vein; HCV: hepatic cardialis venula; OPVR: obliterated portion of venous rings; BD: bile duct; P: pancreas; VA: venae advehentes; VR: venae revehentes; ADPUV: anterior detached portions of umbilical vein. Images from the collection of Dr Anca-Maria Istrate-Ofiţeru.

The venous system starts to develop in a 4-week embryo, from three symmetric paired veins: the umbilical veins (UVs) which drain the chorion, vitelline veins (VVs) draining the yolk sac, and cardinal veins (CVs) which drain the body of the embryo. The fetal liver starts to develop in the septum transversus and allows the connection between the VVs and UVs with the sinusoids. The right umbilical vein (UV) and the left cranial segment of the left UV will atrophy, and the left UV becomes the main pathway of the blood from the placenta [1]. The primitive VVs develop within the liver to form the future portal venous system (PVS). Proximally, VVs will degenerate on the left side, and distally, on the right side, so that the VVs present an S-shaped course around the back and on the front of the gut. Concomitantly, the VVs are at the origin of the superior mesenteric and splenic veins development, which ultimately join into the hepatic portal vein [1] (Figure 1).

By the end of the 10th week of gestation (menstrual age), the PVS is already formed. However, the small size of the embryo does not allow the ultrasound evaluation of this system. Within the liver there are two venous systems: an afferent and an efferent system. The efferent venous system consists of three hepatic veins. The afferent system includes the PVS, which collects blood from the gut, and the umbilical system bringing blood from the placenta to the liver. It has been shown that portal blood supplies exclusively the right hepatic lobe, while the umbilical venous blood supplies both hepatic lobes and the ductus venosus (DV) [1].

The extrahepatic portal vein-main portal vein (MPV) is formed by the confluence of the splenic and superior mesenteric veins. It runs behind the duodenum and drains into the portal sinus near the point of origin of the right intrahepatic portal vein. The right portal vein has two ramifications, the anterior and posterior branch, and the left portal vein has three branches, the inferior, middle, and superior. The portal sinus (PS) is an L-shaped vascular space which extends from the point of origin of the inferior left portal vein (LPV) to the point of origin of the right portal vein (RPV). Thus, it connects the RPV and LPV perfusing the right and left hepatic lobes. Almost all the blood from the MPV is directed to the right hepatic lobe [1,2].

Scanning the fetal portal system is not of outmost importance because of the rarity of its congenital total agenesis. However, there are situations that may associate this condition, which is important for the outcome. The importance of early assessment of the fetal PVS resides from several important considerations. FT detection of agenesis of ductus venosus is possible and important, given its high association with major abnormalities, including portal system anomalies [3]. The development of the PVS was found as an important outcome indicator for agenesis of ductus venosus cases. Besides being a prognostic factor for vascular abnormalities, there are important implications of PVS anomalies in infants regarding metabolic disorder such as high blood galactose. Moreover, during adulthood, the absence of hepatotrophic factors may manifest as acute liver failure or liver nodes, portosystemic encephalopathy, portopulmonary hypertension, and hepatopulmonary syndrome [4,5,6,7].

Based on these considerations, the recognition of PVS anomalies in pregnancy is very important. As stated in the literature, it is preferable an early diagnosis of fetal abnormalities that may lead to an elected pregnancy termination, considering medical, socio-economical, and emotional rationales. Due to important technological progress in ultrasound, with high-resolution probes, and high definition Doppler imaging, an earlier and better visualization of fetal anatomy is achievable, even in the first trimester.

To the best of our knowledge, PVS has been investigated only during the second half of pregnancy [8,9,10,11]. Thus, in this study, we aimed to assess the ability of FT (FT) screening in the evaluation of the PVS and to describe how the detection rate may be affected.

### Aim

The main aim of this study is to prove the feasibility of the FT ultrasound scan to evaluate the fetal PVS anatomy. The second objective of the study is to correlate the ultrasound planes with the correspondent microscopic evaluation as an audit of the early ultrasound findings.

## 2. Materials and Methods

This was a prospective study conducted in the regional tertiary center of Craiova (Prenatal Unit of University Emergency County Hospital Craiova) and MEDGIN-GINECHO Clinic, between January 2020 and September 2020, following the approval of institutional ethics committees. All women provided written informed consent prior to the ultrasound examination.

Participants. FT cases admitted for anomaly scan between 12 and 13.6 weeks of gestation (WG), were considered eligible for the study. They were included in the study consecutively, depending on the availability of the US operators involved. Cases planned for elective abortion, multiple pregnancies, cases that did not comply with the protocol and lost for follow-up pregnancies, that could not be reexamined in the second trimester were excluded from the study.

Ultrasound evaluation. During the standard FT anomaly scan protocol, [12] we investigated systematically the presence and normalcy of PVS features in the transverse plane of the fetal abdomen, using color and power Doppler technique, with a similar approach as in the second trimester. Previously, we proved this technique as very effective in detecting fetal cardio-vascular structural features, including the abdominal venous system [3]. The evaluation was targeted to identify the hepatic course of the umbilical vein (UV), the L-shaped portal sinus (PS), the junction of the PS with the main portal vein (MPV), and left portal vein. Ultrasound examinations were started transabdominally. Transvaginal approach, seriated evaluations in the same day, or a rescheduled appointment was carried out when the examination conditions were unfavorable (persistently unfavorable fetal position or unfavorable maternal conditions). Voluson E10 and an E8 (General Electric System, GE Healthcare, Zipf, Austria) ultrasound machines, were used, equipped with a 4–8-MHz curvilinear and a RIC5-9-D transducers.

The patients were equally divided in two matched groups, in terms of gestational age, BMI, and uterine retroversion, fibroids and abdominal scar. Group I was scanned by two sonographers with extensive experience in fetal ultrasound morphology and maternal–fetal medicine, and Group II was examined by two sonographers with three years of experience in obstetrics and gynecology ultrasound examination. Prior to ultrasound scans, all 4 examiners participated to a series of meetings where the embryology of the fetal umbilical-portal venous system and Doppler evaluation of small vessels were discussed.

All cases scanned in the first trimester were followed in second trimester and the anatomic features were reconfirmed.

Maternal characteristics and medical history were recorded in all scanned pregnant women.

Pathology evaluation. Necropsy has been performed in 3 FT cases and in 3 s trimester cases in order to evaluate the potential of macroscopic and microscopic means to audit fetal PVS features in relation to the ultrasound findings. The necropsy of the specimens was performed in the laboratory of Pathology Clinic of the University Emergency County Hospital Craiova and the liver was removed in bloc with the rest of the abdominal organs. The macroscopic and microscopic evaluation of the liver was performed in the Research Centre for Microscopic Morphology and Immunology University of Medicine and Pharmacy of Craiova.

Second trimester cases were first evaluated macroscopically, by examining the vascular system appearance of seriate axial slices of the fetal liver. In FT cases, macroscopic evaluation was not attempted, given the small size of the fetal liver and targeted vascular system, and previous experience of our center. The whole liver was fixed in 10% neutral buffered formalin and processed for paraffin embedding (FFPE). A HMB350 microtome equipped with a section transfer to water bath system (STS microM) performed the serial section of the block at 5 μm. The sections were stained classically, using hematoxylin-eosin (HE), and immunohistochemically, using antibody anti-alpha smooth muscle actin (αSMA), anti-cluster of differentiation 68 (CD68), and anti-cytokeratin AE1-AE3 (Table 1). The slides were deparaffinized, rehydrated with successive alcohol baths with decreasing concentration of 100%, 96%, 90%, 70% (5 min each) and then with distilled water (dH_2_O) for 15 min. After the rehydration with alcohol and distilled water the nuclei were labeled with hematoxylin and the cytoplasm of the cells with eosin. After staining, the slides were fixed with Canada balsam. The immunohistochemical procedures involved antigenic exposure which was performed using ethylenediaminetetraacetic acid (EDTA) pH 9 or with citrate pH 6. This technique was followed by endogen peroxidase deactivation with the aid of 3% oxygenated water (H_2_O_2_) (30 min), non-specific endogen situses blockage with skim milk (30 min). Following these procedures, the primary and the secondary antibodies were applied. After the primary antibody, the slides were kept at 4 °C (for 18 h). The following day, second antibody was applied (mouse/rabbit immunoglobulin G (IgG) antibody, VC002- 025, R&D Systems, VisUCyte Horseradish peroxidase (HRP) Polymer) (one hour). The aid of 3,3′-diaminobenzidine (DAB) (Dako) allowed the development of the slides and the nuclei were labeled with hematoxylin solution. As a final procedure, the slides were dehydrated with increasing concentration of alcohol 70%, 90%, 96%, 100% (5 min each), clarified in three successful xylene baths (3 × 15 min/bath). Moreover, a slide was fixed on the tissue using Canada balm. The colored slides on which we could identify the PVS and the UV, were scanned with a Motic EasyScan device (Motic China Group Co., Ltd., Xiamen, China), at a 20× lens, and saved in proprietary format in a database in the Motic Digital Slide Assistant package. They were then exported as tiff files at full resolution for further processing.

## 3. Results

**Figure 2:**
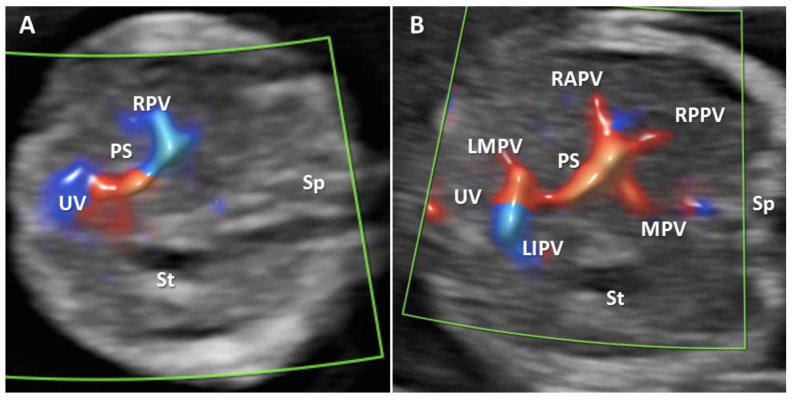
Transverse plane of the FT fetal abdomen, with high-definition directional power Doppler applied. (**A**): Transabdominal approach demonstrating the normal L-shaped UV confluence. (**B**): Transabdominal approach demonstrating all PVS features. MPV main portal vein, PS portal sinus, UV umbilical vein, RAPV anterior branch of right portal vein, RPPV posterior branch of right portal vein, LIPV left portal vein inferior branch; LMPV left portal vein medial branch, St stomach, Ao aorta, Sp spine.

**Figure 3:**
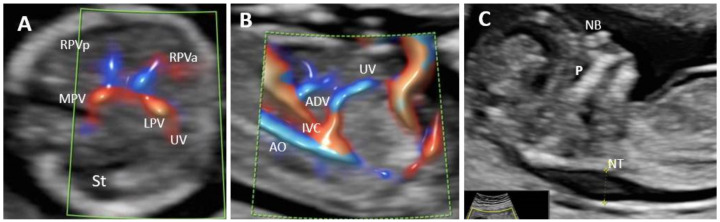
Agenesis of ductus venosus (ADV) in a first trimester case with umbilical vein drainage into inferior vena cava (IVC) and increased nuchal translucency. (**A**): Transverse plane of the FT fetal abdomen, with high-definition directional power Doppler applied. An ”H”-shaped variant of the intrahepatic portal veins connection is identified; (**B**): high-definition directional power Doppler in the sagittal plane of the fetal abdomen (same case) showing ADV with umbilical vein drainage into the inferior vena cava; (**C**): mid-sagittal view of the fetal face with the measurement of the thickened NT. MPV main portal vein, St stomach, LPV left portal vein, UV umbilical vein, RPVa anterior branch of right portal vein, RPVp posterior branch of right portal vein, Ao aorta, IVC inferior vena cava, ADV ductus venosus agenesis, P palate, NB nasal bone, NT nuchal translucency.

**Figure 4:**
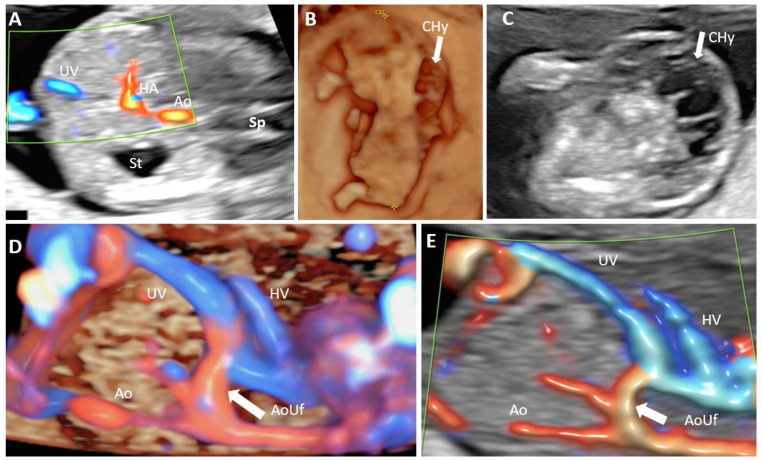
Absence of the portal system in a first trimester case associated with hygroma and aorto-umbilical fistula. (**A**): Transverse plane of the upper abdomen with color Doppler applied, showing umbilical cord insertion, stomach, the prominent hepatic artery and no afferent liver venous perfusion; (**B**): midsagittal plane reconstructed from a three-dimensional volume acquisition were the crown-rump length is measured and fetal cystic hygroma can be observed (white arrow); (**C**): transverse sonographic view of the neck showing the septated nuchal cystic mass (white arrow); (**D**): 4D STIC showing in the longitudinal view of the fetal abdomen an abnormal connection (white arrow) between umbilical vein and aorta. (**E**): same aspects as (**D**), using two dimensional color Doppler assessment. UV umbilical vein, HA hepatic artery, Ao aorta, St stomach, Sp spine, CHy cystic hygroma, AoUf aorto-umbilical fistula.

Total of 100 cases were targeted for examination in each group. The targeted gestational age was 12 + 4 to 13 + 0, which was achieved in 175 patients (87.5%). The acceptability for the supplemental evaluation of the PVS in the FT was 98%. Group I. Evaluation of all PVS features by TA approach was possible in 27% of the 100 cases (Figure 2B). The unfavorable conditions for this low rate were indicated as follows: BMI greater than 24 in 26% cases, unfavorable fetal position in 12.32% cases, retroverted uterus in 12.32% cases, abdominal scar in 10.96% cases, and fibroids in 4.11% cases. A combination of unfavorable conditions was reported in one-third of the cases (34.23%): increased BMI and abdominal scar 16.43% cases, unfavorable fetal position and increased BMI in 12.32% cases, fibroids and abdominal scar in 5.48% cases. The visualization rate was much higher for the identification of only the L-shaped UV confluence-91% cases (Figure 2A). For the 73 cases in which all the PVS features were not visualized by TAUS, TVUS, reevaluation after several days, or a combination of the two of them, associated a satisfactory visualization rate of 83.56% cases. The main associate unfavorable condition at reschedule and TVUS was a persistent unfavorable fetal position: vertical lie, in 5 cases (41.66%) and when the fetus was far from the probe, 6 cases (50%). Isthmic fibromyoma hindered the evaluation of PVS features in one case (8.33%). The concomitant transvaginal approach or reevaluation after several days allowed a final visualization rate of 88% cases when the aim of the scan was to evaluate all PVS feature. The visualization of the L-shaped UV confluence achieved a rate of 98% of the cases. (Table 2). In Group I we identified one case with an atypical H-shaped type portal venous confluence which associated ductus venosus agenesis (ADV) with umbilical vein drainage into inferior vena cava (IVC) and increased nuchal translucency (5 mm) (Figure 3). Another abnormal finding in this group was a case of total portal venous system agenesis (TPVSA) which associated aorto-umbilical fistula and cystic hygroma (Figure 4, white arrow). Between the two sonographers there were no significant differences in the satisfactory evaluation rate of the PVS (92% versus 94%).

In Group II was evaluated the same number of FT pregnancies but the visualization rate was lower. All PVS features were successfully TA visualized in 14% cases. The unfavorable conditions that lead to a low rate were combined as follows: BMI greater than 24 (25.58%), unfavorable fetal position (19.76%), retroverted uterus (18.60%), fibroids (6.97%), abdominal scar (4.6%). In some cases, there were reported associations of these conditions: unfavorable fetal position and increased BMI (20.93%), fibroids and abdominal scar (3.49%). The L- shaped UV confluence was visualized in 79% cases.

In 86 remained cases, where TAUS could not visualize all PVS features, TVUS or rescheduling, allowed to evaluate all PVS features in 67.44% cases; in the rest of the cases in which the sonographers did not evaluated all PVS features, 2 cases associated low-lying fibroids of the uterus and in 26 cases the fetal position was persistent unfavorable for the examination. For the 100 cases, the final visualization rate of all PVS features was 72% and 95% for the L-shaped UV confluence visualization.

When the sonographers chose to reschedule the appointment to complete the examination, the visualization rate increased in both groups as the position of the fetus became more favorable. (Table 2).

**Figure 5:**
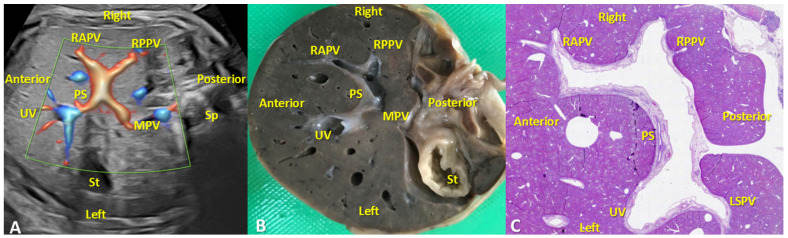
Transverse planes at the level of PVS in the second trimester of pregnancy. (**A**):Transverse plane of the upper abdomen with color Doppler evaluation, showing hepatic course of the umbilical vein (UV), the L-shaped portal sinus (PS), the junction of the PS with the main portal vein (MPV), the left portal vein, the right portal vein and its branches. (**B**): Normal macroscopically appearance of the liver demonstrating the PS, MPV, UV, LSPV, RAPV, RPPV. St-stomach. Sp- spleen. PS: portal sinus, MPV: main portal vein, UV: umbilical vein, LSPV: left portal vein and the superior branch, RAPV: right anterior portal branch, RPPV: right posterior portal branch. (**C**): Transverse section in the hepatic parenchyma which allowed the identification of the PS, UV, LSPV, RAPV, RPPV. Classic hematoxylin-eosin staining, ×100.

In order to evaluate the components of the PVS in the first two trimesters, we performed transverse section of the fetal upper abdomen at the level of PS in pathology specimen of 3 FT normal fetuses, and in 3 normal second trimester fetuses. The second trimester autopsy offered a sufficient macroscopic visualization of PVS, which allowed proper correlation with US appearance. In the second trimester specimens, serial cross-sections from the diaphragmatic aspect of the liver to its visceral face, in a cranio-caudal direction, allowed for the macroscopic identification of the umbilical vein (UV), main portal vein (MPV), portal sinus (PS), left portal vein (LPV), and right portal vein (RPV) (Figure 5).

**Figure 6:**
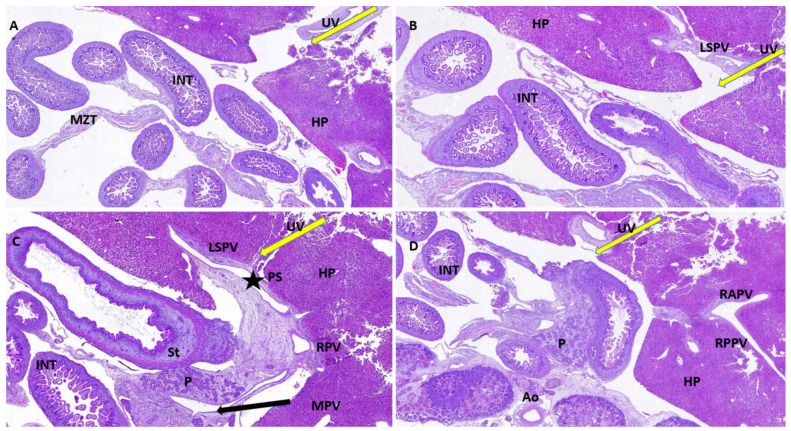
Histological appearance of the FT liver with normal PVS. (**A**): Histopathological aspect of FT fetal liver. It is observed how UV enters the hepatic structure (yellow arrow) and reaches its visceral surface near the intestinal loops; (**B**): LSPV is identified in the structure of the liver parenchyma, toward its posterior face, in the vicinity of the intestinal loops and the stomach; (**C**): the L-shaped umbilical vein confluence and the PS (star) is observed in the structure of the liver parenchyma; (**D**): the two main branches of the RPV are identified as RAPV and RPPV. Classic hematoxylin-eosin staining, ×100. UV: umbilical vein, HP: hepatic parenchyma, INT: intestines, MZT: mesentery, LSPV: left superior portal vein, PS: portal sinus, RPV: right portal vein, MPV: main portal vein, P: pancreas tissue, St: stomach, Ao: aorta, RAPV: right anterior portal vein, RPPV: right posterior portal vein.

**Figure 7:**
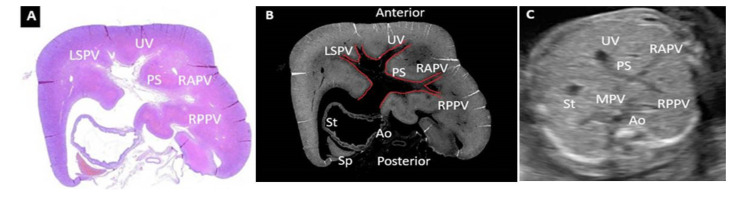
Histopathological and ultrasound aspect of normal FT liver. (**A**): Normal histological appearance of the FT liver, demonstrating the UV, LSPV, PS, RAPV, RPPV. Classic hematoxylin-eosin staining, ×100; (**B**): image processed in negative format which highlights more strongly the elements described in image (**A**); (**C**): transverse view of the fetal abdomen on grey scale showing the UV, MPV, PS, RAPV, RPPV, St, Ao. UV: umbilical vein, LSPV: left superior portal vein, PS: portal sinus, RAPV: right anterior portal vein, RPPV: right posterior portal vein, St: stomach, Ao: aorta, Sp: Spleen.

**Figure 8:**
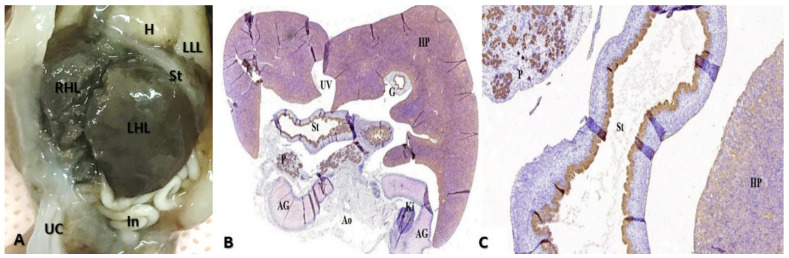
Anatomic and histopathological aspects of FT organs. (**A**): Anatomopathological specimen showing fetal liver which occupies most of the abdominal cavity. (**B**,**C**): Transverse section at the level of the abdomen. We can identify: the hepatic parenchyma located anteriorly, with the disorganized hepatocyte cords, weakly immunolabeled with CK AE1-AE3, the stomach (St) at which the gastric epithelium was intensely immunolabeled, the pancreas (P) with immunoreactive acinar cells, the two adrenal glands (AG) and one kidney (Ki). Moreover, part of gallbladder (G) are immunolabeled. Immunohistochemical staining with anti-CK antibody AE1-AE3; (**B**) the hepatic parenchyma (HP) is observed, with the disorganized hepatocyte cords, weakly immunolabeled with CK AE1-AE3, the stomach (St) with the gastric epithelium intensely immunolabeled, the pancreas (P) with immunoreactive acinar cells. Immunohistochemical staining with anti-CK antibody AE1-AE3. CK: Cytokeratin, HP: hepatic parenchyma, UV: umbilical vein, G: gallbladder, St: stomach, AG: adrenal glands, Ao: Aorta, Ki: kidney, In: intestine, UC: umbilical cord, RHL: right hepatic lobe, LHL: left hepatic lobe, LLL: left lower lobe of the lung.

**Figure 9:**
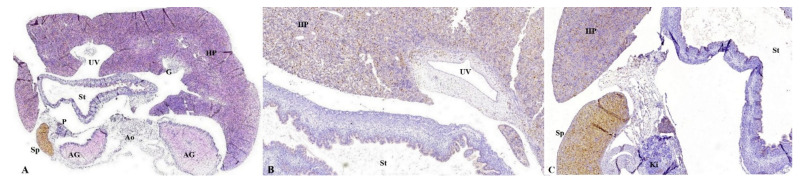
Transverse section at the level of the abdomen. (**A**): The hepatic parenchyma (HP) located anteriorly, the disorganized hepatocyte cords, with the presence of Kuffer cells among the hepatocyte cords, the stomach (St) with macrophages in the gastric wall, the posterior adrenal glands (AG) and the spleen (Sp), intensely immunolabeled with anti-CD68, which demonstrates the increased number of the macrophages present in the tissue structure. Immunohistochemical staining with anti-CD68 antibody; (**B**): the hepatic parenchyma (HP) located anteriorly, the disorganized hepatocyte cords, with the presence of Kuffer cells among the hepatocyte cords, the stomach (St) with macrophages in the gastric wall; (**C**): the hepatic parenchyma (HP) anteriorly, disorganized hepatocyte cords, with the presence of Kuffer cells among the hepatocyte cords, the stomach with macrophages in the gastric wall, the posterior kidney (Ki) located posteriorly and the spleen (Sp) on the left, intensely immunolabeled with anti-CD68, which demonstrates increased number of macrophages present in the tissue structure. Immunohistochemical staining with anti-CD68 antibody. CD: Cluster of differentiation, HP: hepatic parenchyma, UV: umbilical vein, G: gallbladder, P: pancreas, St: stomach, AG: adrenal glands, Ao: Aorta, Sp: spleen, Ki: kidney.

**Figure 10:**
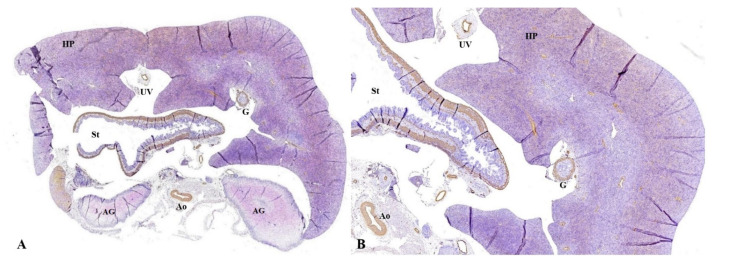
Transverse section at the level of the abdomen. (**A**): The liver parenchyma (HP) located anteriorly, the disorganized hepatocyte cords, the muscular tunics of the blood vessels immunolabeled with brown in both the liver parenchyma and the great hepatic vessels, the muscular and the middle tunic of the stomach (St), muscular tunic of the aorta (Ao); (**B**): the hepatic parenchyma, disorganized hepatocyte cords, muscle tunics of the blood vessels immunolabeled with brown in both the hepatic parenchyma (HP) and the great hepatic vessels are observed. Moreover, the muscular and the middle tunic of the stomach (St), muscular tunic of the aorta (Ao) are identified. Immunohistochemical staining with anti-αSMA antibody. αSMA: alpha actin smooth muscle, HP: hepatic parenchyma, UV: umbilical vein, G: gallbladder, St: stomach, AG: adrenal glands, Ao: Aorta, Sp: spleen.

As stated before, the FT liver specimens have been further processed for paraffin embedding in order to microscopically evaluate the PVS (Figure 6 and Figure 7). Serial sections through the fetal abdomen showed the anatomical location of the fetal organs, but also certain tissue features, present since fetal life. With the help of CK AE1-AE3 we highlighted the Remack cords of the liver parenchyma, but also the gastric epithelium, gallbladder epithelium, and intensely immunoreactive pancreatic acinar cells (Figure 8). The topography of the FT fetal organs is similar to the one met in the second trimester. CD68 highlighted the presence of macrophages in the structure of the gastric wall, but also the existence of Kuffer cells since the first trimester. The most intense immunoreactivity was in the spleen (Figure 9). The anti-αSMA antibody immunolabeled the muscle tunics of the blood vessels in both the liver parenchyma and the great liver vessels. It also immunolabeled the muscular and the middle tunics of the stomach, muscular tunic of the aorta, located posteriorly (Figure 10).

## 4. Discussion

The use of ultrasound in pregnancy has been extensively studied and its applicability has been shown for early diagnosis of fetal abnormalities. The main purpose of prenatal diagnosis of fetal anomalies is to provide an accurate prognosis for the couple, as early as possible during pregnancy. The agenesis of the PVS represent a well-known prognostic factor in cases of agenesis of ductus venosus [10]. Further, the identification of a normal PVS is important, as the complete agenesis of PVS is associated with severe health problems during the postnatal life [13]. Anomalies of PVS occurs as TPVSA when the splenic and superior mesenteric veins do not form the MPV or, if they do, the drainage is directed into the systemic circulation. TPVSA frequently associates other fetal anomalies: heterotaxy–polysplenia, congenital heart defects, or chromosomal anomalies, while partial portal venous system agenesis (PPVSA), usually represented by the agenesis of the right portal vein, is more frequently diagnosed [11] and rarely associates other malformations. The pediatric and gastroenterology literature suggest that PPVSA may have an almost normal outcome but neonatal and childhood evaluations are needed to detect the postprandial ammonia levels, liver nodes, high blood galactose, hemodynamic deterioration [11,14]. Contrary to PPVSA, where any remnant of portal vein branches allows a favorable prognosis, TPVSA cases seems to have a bad outcome, given its high association with major abnormalities [10,15].

This discussion is very important in the context of FT evaluation. The features of the PVS were completely visualized in a minority of the cases evaluated by standard approach, transabdominally. However, the L-shaped confluent of the UV and its normalcy was visualized in the vast majority, regardless the sonographer’s experience. Therefore, TPVSA, which represent the major anomaly of the portal system should be rarely missed, if this item is part of the check-list. PPVSA cannot be detected based on this parameter, but its clinical importance appears to be minor.

The experience of the sonographers is important. TAUS performed by the experienced operators, successfully evaluated all PVS features in one quarter of the cases, while those elements were visualized in only 14% of the cases examined by less experienced operators. However, the normality of L-shaped UV confluence, which excludes major PVS abnormalities, was visualized transabdominally in 91% and 79% of the cases by the experienced and less experienced sonographers, respectively.

The main conditions reported by the examiners that hindered the visualization of the PVS features were represented by an increased BMI and unfavorable fetal position. Obesity has doubled since the 1980s, According to WHO, the ultrasound evaluation of fetal structures is affected by maternal obesity [16,17]. Our data among the high BMI cases confirms the published data [18]. In both groups, the evaluation of all PVS aspects among the women with BMI greater than 24 was possible only by using the transvaginal approach.

Reschedule and TVUS offered valuable information, especially for the proper evaluation of all PVS features. This approach was useful to identify PVS features in more than four-fifths (83%) and more than two-thirds (67%) of the cases not satisfactorily evaluated transabdominally. Regarding the evaluation of the L-shaped UV confluence, 7 more cases were properly visualized in group I and 16 in group II. We do not suggest a routine transvaginal evaluation to identify the features of portal system when they are not apparent transabdominally, but only highlight the potential of reevaluation and TVUS scan for early visualization of the portal system characteristics.

The final visualization rate of PVS features was 88% for experienced sonographers and 72% in the group of less experienced sonographers. Visualization of a UV confluence presented much higher final rates of visualization—98% and 95%, respectively. The main finding of our study is that globally, the evaluation rate of all PVS features in the FT is 80%. This proves the ability of early scan to accurately evaluate the components of this system. Although the PV is half the diameter of the UV, its evaluation yielded good detection percentage.

The guidelines suggest that the anatomical assessment at time of FT should include axial view of upper abdomen, showing the stomach present in left upper quadrant [18]. There are no recommendation in evaluating the PVS in the first or second trimester, therefore the anomalies are largely underdiagnosed at the time of fetal anomaly scan. However, our study showed that, the early detection of major portal anomalies is possible (Figure 4).

Based on our experience, we observed that the macroscopic evaluation of PVS is feasible only in the second trimester, due to the small size of the liver in FT and the friability of this tissue. Instead, the FT the histological evaluation of the liver appears to be a good audit in confirming the PVS features in the FT. The orientation of the fetal liver is extremely important to capture the sections in the PVS plane. This is why, usually the evaluation of this system requires the study of serial microscopic sections, which display all the elements described above (Figure 6).

Microscopically, in the first trimester of pregnancy and beyond, the liver consists of a variety of cells such as hepatocytes and hematopoietic line cells and is formed by modeling the endoderm [19]. Those elements were also highlighted in our study. The cells of the hematopoietic line are not formed by the endoderm but by the mesoderm, and will colonize the liver by migration [20]. Identifying the structure of hepatocyte cords, immunolabeled with CK AE1-AE3, facilitates the delimitation of the PVS. Kupffer cells and Ito cells are known to play an important role in the proper development of the adult liver.

Through serial histological sections we observed parenchymal cells, hepatocytes, which are placed in Remack cords, but they are disorganized, compared to the adult liver, when they converge centrally, to the centrolobular vein [21,22,23]. We detected these hepatocyte cells using anti-CK antibody AE1-AE3, which represents a pan-cytokeratin, a cocktail formed by cytokeratins 1–8, 10, 14–16, and 19. [24] Those hepatocytes are stained weakly compared to the gastric epithelium, gallbladder epithelium or to the pancreatic acinous serous structures. The previous elements are highlighted in the histological sections, marked intensely immunohistochemically. We also identified in the FT liver the presence of non-parenchymal cells, which constitute about 40% of the total number of liver cells [24]. Hepatic sinusoidal capillaries, similar to those of the mature liver, present squamous endothelial cells, as well as phagocytic Kupffer cells, immunolabeled with the anti-CD68 antibody [25].

Identifying Kupffer cells in FT pregnancy emphasizes the importance that they have in removing foreign structures or particles that reach the liver through the portal system or to pinocytosis smaller particles [26]. Moreover, these cells are very important in achieving innate immune responses, in the metabolism of compounds such as lipids, small particles, or protein complexes and in eliminating cellular debris from the circulation. These cells have the ability to proliferate and regenerate on their own, in contrast to the macrophages arising from circulating monocytes. The most immunologically active cells are the Kupffer cells from zone 1 of the liver lobes, as they are more exposed to substances stored through the hepatic intravenous system [27,28].

Our findings have shown that Stellar, non-parenchymal cells, Ito, located in the perisinusoidal space–Disse [29], were present in the FT liver. We demonstrated that those cells are active since FT, as the anti-αSMA antibody marked the actin myofilaments present intracellularly. These stellate cells represent 5–8% of all liver cells in adults [30]. The function and role of these resting cells are not fully studied, but studies have shown that they behave like an antigen-presenting cell, stimulating the action of natural killer (NK) cells [31]. When activated, these cells can proliferate, show contractility and chemotaxis, producing hepatic extracellular matrix and excess collagen [32].

In FT intrauterine life the liver occupies most of the abdominal cavity, aspects highlighted in the histology images previously exposed (Figure 8). The liver grows rapidly from the fifth to the tenth week of intrauterine life, occupying most of the upper abdomen and weighing about 10% of the total body weight in the 10th week of evolution. At this stage, the liver does not fulfill the functions of digestion and filtration, these functions are performed by the placenta, through the UV. Liver development and segmentation are determined by the oxygenated blood brought in by UV. UV is very well represented since the FT of pregnancy, as demonstrated in Figure 7 and Figure 8. The histological aspect of UV is marked in the images presented above, observing its three tunics (vascular endothelium, middle tunic, muscle, marked with anti-αSMA antibody, and the outer adventitia formed by loose connective tissue) (Figure 10).

The originality of our study consists in the complex ultrasound and pathological evaluation of the portal venous system in the first trimester. After a careful search in the literature, we observed that the PVS was evaluated only in the second half of the pregnancy [9,10,15,18,33]. The VVs anomalies have not been analyzed in the FT studies. On the other hand, contrary to the UV anomalies, as persistent right umbilical vein, umbilical vein varix, which are frequently reported, the VV anomalies are rarely identified and underdiagnosed, and only a few cases have been reported in the second trimester fetuses [34].

The limitation of our study is due to the rarity of the PVS anomalies. Thus, reliability and accuracy of the early US scan could not be calculated, given the low number of such malformations. Further larger studies are needed for these statistical issues.

## 5. Conclusions

The key finding of this paper shows that during FT anomaly scan, even unexperienced sonographers can evaluate the integrity of the PVS in the large majority of cases.

The sonographers experience, pregnant women BMI and uterine anomalies as fibroids or retrovestion represents important circumstances that affect the rate of visualization, but combining the abdominal with vaginal approach and reexamination, a global rate of 80% accurate scans can be achieved. Toward the end of the FT, the visualization of a normal L-shaped UV confluence, that excludes major PVS abnormalities, is achievable in almost all cases, indifferently the examiners experience. The sonographers experience, pregnant women BMI, unfavorable fetal position, and uterine anomalies as fibroids or retroversion represents important circumstances that affect the rate of visualization, but combining the abdominal with vaginal approach and reexamination, a global rate of 80% visualization rate of the L-shaped UV confluence can be achieved, indifferently the examiners experience, that enables the detection of major PVS abnormalities. The transabdominal visualization of all PVS features presents a low rate even in expert hands (27%) and even lower (14%) in less experienced sonographers. However, rescanning and use of transvaginal ultrasound (TVUS) significantly increase visualization rates to 88% and in 72%, respectively.

In the second trimester of pregnancy the audit of the ultrasound examination of the portal system can be performed macroscopically on the autopsy specimens and microscopy is helpful as an adjunct of the pathology evaluation. However, in the FT, the audit can only be performed microscopically, with relatively little resources involved and good results.

Our paper provides additional clinical insight into early prenatal ultrasound evaluation of PVS, that may serve professionals for counselling in pregnancies where anomalies of the portal system or ductus venosus are suspected.

## Figures and Tables

**Table 1:** Immunohistochemical panel of antibodies used in the study.

Antibody	Manufacturer	Clone	Antigenic Exposure	Secondary Antibody	Dilution	Labeling
Anti-CK AE1-AE3	Dako	AE1/AE3	Citrate, pH 6	Monoclonal Mouse Anti-Human Cytokeratin	1:50	Epithelial tissues
Anti-CD68	Dako	KP1	Citrate, pH 6	Monoclonal Mouse Anti-Human CD68	1:100	Macrophages
Anti-αSMA	Dako	1A4	Citrate, pH 6	Monoclonal Mouse Anti-Human Smooth Muscle Actin	1:100	α actin smooth muscle

CK: cytokeratin; CD: cluster of differentiation; αSMA: alpha actin smooth muscle.

**Table 2:** The comparative satisfactory evaluation rate of the PVS items in the two groups, reasons for not-satisfactory visualization and the results of alternative approaches.

	Identification of All PVS Features by TAUS	Associated Unfavorable Conditions	Visualization of the L-Shaped UV Confluence, TA US	Satisfactory Visualization of PVS Features after Reschedule/TV Evaluation	Associate Unfavorable Conditions at Reschedule/TV Evaluation	Visualization of the L-Shaped UV Confluence at Reschedule/TV Evaluation	Final Visualization Rate of PVS Features	Final Visualization Rate of L-Shaped UV Confluence
Group I	27 (of 100 cases), 27%	Fibroids 3 cases, 4.11%	91 (of 100 cases) 91% (one atypical, one abnormal-TPVSA)	61 (of 73 cases), 83.56%	persistent unfavorable fetal position- vertical lie, 5 cases 41.66%)	7 of 9 cases (77.77%)	88%	98%
		BMI > 24 19 cases, 26.02%						
		unfavorable fetal position 9 cases, 12.32%						
		retroverted uterus 9 cases, 12.32%			persistent unfavorable fetal position- far from the probe 6 cases (50%)			
		Abdominal scar 8 cases, 10.96%			Istmic fibromyoma 1 case (8.33%)			
		Fibroids and abdominal scar, 4 cases, 5.48%						
		Unfavorable fetal position and increased BMI, 9 cases, 12.32%						
		Increased BMI and abdominal scar, 12 cases, 16.43%						
Group II	14 (of 100 cases), 14%	Fibroids 6 cases, 6.97%	79 (of 100 cases), 79%	58 (of 86 cases), 67.44%	low-lying fibroids 2 cases, 7.14%	16 of 21 cases (76.19%)	72%	95%
		BMI > 24 22 cases, 25.58%						
		unfavorable fetal position 17 cases, 19.76%			persistent unfavorable fetal position -vertical lie, 12 cases, 42.85%			
		retroverted uterus 16 cases, 18.60%			persistent unfavorable fetal position- fetus far from the probe 14 cases, 50%			
		Abdominal scar 4 cases, 4.65%						
		Fibroids and abdominal scar, 3 cases, 3.49%						
		Unfavorable fetal position and increased BMI, 18 cases, 20.93%						

TA: transabdominal approach; TV: transvaginal approach; BMI: body mass index; US: ultrasound.

## Data Availability

Not applicable.
